# Barriers to Accessing and Negotiating Mental Health Services in Asylum Seeking and Refugee Populations: The Application of the Candidacy Framework

**DOI:** 10.1007/s10903-019-00929-y

**Published:** 2019-08-23

**Authors:** Catharina F. van der Boor, Ross White

**Affiliations:** Institute of Life and Human Sciences, University of Liverpool, Brownlow Hill, Liverpool, L69 3BX UK

**Keywords:** Asylum seekers, Refugees, Mental health services, Candidacy framework

## Abstract

This review brought together research investigating barriers asylum seekers and refugees (AS&R) face in accessing and negotiating mental health (MH) services. The candidacy framework (CF) was used as synthesizing argument to conceptualize barriers to services (Dixon-Woods et al. in BMC Med Res Methodol 6:35, 2006). Five databases were systematically searched. Twenty-three studies were included and analyzed using the CF. The seven stages of the framework were differentiated into two broader processes—access and negotiation of services. Comparatively more data was available on barriers to access than negotiation of services. The *Identification of Candidacy* (access) and *Appearances at Services* (negotiation) were the most widely discussed stages in terms of barriers to MH care. The stage that was least discussed was *Adjudications* (negotiation). The CF is useful to understand inter-related barriers to MH care experienced by AS&R. A holistic approach is needed to overcome these barriers together with further research investigating understudied areas of candidacy.

## Introduction

### Asylum Seekers, Refugees and Mental Health

Increasing numbers of people are leaving their homelands because of human rights violations, persecution and conflict. By June 2018, there were an estimated 68.5 million forcibly displaced individuals worldwide of whom 3.1 million were classified as asylum seekers, and 25.4 million as refugees [1]. The arrival of such high numbers of asylum seekers and refugees (AS&R) places substantial pressures on host countries and their services, including mental health (MH) care systems [2, 3].

AS&R can be subject to pronounced stressors and adverse conditions pre-migration, during migration and/or post-migration (i.e. [4–6]). In light of potential exposure to these stressors, it is perhaps unsurprising that AS&R show higher rates of post-traumatic stress disorder (PTSD) than host populations [5, 7, 8]. Indeed, PTSD rates have been noted to be almost 10 times more frequent in AS&Rs than in age-matched host populations [9]. The literature shows there is high variability in the studied prevalence rates of mental disorders in AS&R populations, compared to host populations. Bogic et al. [7] also found that refugee samples are likely to have high prevalence rates of depression, which often exceed those reported by samples in host nations. However, a review conducted by Priebe et al. [5] concluded that the rates of mood, psychotic, and substance-use disorders found in AS&Rs groups are within the range of the rates present in host groups. Although the evidence base for prevalence rates varies, the literature shows that the exposure to adverse events can have a negative impact on the MH of AS&R. Given the high absolute numbers of AS&Rs moving across borders, this can constitute a significant challenge to healthcare systems in receiving countries.

### The Candidacy Framework: ‘Accessing’ vs. ‘Negotiating’ Care

Research suggests that despite this increased vulnerability, there is often an underrepresentation of AS&Rs in the health care (HC) services [10, 11]. Identified challenges to accessing services include social, linguistic, economic, clinical severity, and cultural differences in symptom presentation, as well as systemic discrimination [10, 12, 13]. There is also evidence that legal entitlement; formal access to care regulations and the migration process inhibit access in various high-income countries [3]. All individuals have a fundamental legal right to health and to access HC, which is represented both in international and European instruments, such as the European Charter of Fundamental Rights [14]. However, depending on migration status, migrants may have limited entitlements to HC due to national laws and policies [15]. For example, the structure of health systems, which is determined by national policies, can determine the availability of services, the need for HC insurance and the extent of HC coverage, amongst others, which can all impact on the ability to access HC in subgroups of migrants [16].

The candidacy framework (CF) was initially developed as a counter to existing ideas of ‘access’ that draw on data about service utilisation (e.g. number of consultations), but which often fail to capture the complex processes involved in navigating care and fails to account for those who do not seek or are refused services. Dixon-Woods and colleagues summarize *candidacy* as the ways in which eligibility for medical help and intervention is negotiated between individuals and HC services [17]. Candidacy can be understood as a dynamic and contingent process which is constantly defined and redefined through interactions between the individual and professionals. Therefore, people’s previous interactions and experiences with HC services and professionals can also shape an individual’s candidacy [17]. As such, an individual’s identification of their ‘candidacy’ for accessing and negotiating HC services can be culturally, structurally and professionally constructed [18]. This framework provides a means to explore these negotiations and how they can act as barriers to care [17]. The CF proposes seven overlapping stages two of which address immediate access (stage 1 and stage 2) and five which address negotiation (see Table 1).

**Table 1:** The seven stages of candidacy [17]

Stages of candidacy	Description of stages	Examples
1. Identification of candidacy by the individual	Process through which individuals decide that they have a particular need and that assistance may be required	Individuals’ recognition of MH symptoms
2. Navigation	Knowing how to make contact with appropriate services in relation to identified candidacy	Being allowed time off work for appointments
3. Permeability of services	Ease with which people can use services. Includes the level of explicit and implicit gate-keeping within a service and the complexity of its referral systems; in addition, it refers to the ‘cultural alignment’ between users and services	Provision of translational services
4. Appearing at services and asserting candidacy	The work that individuals must do to assert their candidacy in an interaction with a HC professional	The service user feels taken seriously’—‘acknowledged’ and/or ‘understood
5. Adjudications by professional	Refers to the judgments and decisions made by professionals which allow or inhibit continued progression of candidacy	Being referred on to mental health services
6. Offers of, and resistance to, specific services	Emphasizes that follow-up services may be appropriately or inappropriately offered and that these may or may not be acted upon by service-users	Refusal of offer of medication
7. Operating conditions and local production of candidacy	Incorporates factors that influence decisions about subsequent service provision (i.e. the resources available for addressing candidacy) and the kinds of contingent relationships that develop between professionals and service-users over a number of encounters	Adapting the frequency of consultations to the individual’s needs

The CF has thus far mainly been applied in populations whose entitlement to care is relatively stable and comprehensive. Mackenzie et al. have called for the exploration of candidacy in contexts where vulnerable individuals may be subject to compromised services (i.e. lack of citizenship or stigma) [19]. To date, only one study has specifically applied the CF to understanding the help-seeking trajectory of asylum seekers [20]. The study found that asylum seekers’ precarious migratory status constrained their candidacy for obtaining HC. Barriers included having misinformation about HC coverage, tiresome administrative procedures specific to asylum seekers, and long waiting times. The findings showed that migratory status and feelings of marginalization and insecurity that come from their migrant status, appeared to amplify the effects of the barriers to care and even minor difficulties to access could have dramatic effects on future help-seeking behavior [20].

The current review uses the CF to synthesize qualitative research findings investigating barriers to accessing and negotiating MH services for AS&Rs in high-income countries (HIC). The structure and delivery of HC services (including MH services) in HIC are comparatively well resourced and formalized. As such, the exclusive focus on including studies undertaken in HIC in the current review allowed for a fuller examination of barriers and facilitators relating to accessing and negotiating services than including studies conducted in low- and middle-income countries, where services may be non-existent, would have permitted. With HIC-based HC services and providers seeing increasing numbers of AS&R groups [21, 22], there is also need for a more detailed understanding of the barriers to accessing specialist services in HICs. This review is the first to focus specifically on barriers to MH services for AS&R populations by using a CF. The findings of this review can be used to inform the design and delivery of forms of MH support for this underserved population in HIC of resettlement.

## Methods

### Search Strategy

The PsychINFO, Medline, Web of Science, SocINDEX and Embase databases were searched up to December 2018. Each search contained three segments; (1) asylum seekers, refugees and displaced persons, (2) MH services and MH problems and (3) candidacy, see “Appendix A” for an example of the full search strategy for the PsycINFO database. The search strategy used was adjusted to each database using the Kings College London library guide [23]. Additionally, *reference chaining* was completed—a process by which academic papers that have cited an included study are electronically identified and screened for potential inclusion and the reference list of each included study are also searched for studies that could meet eligibility criteria for inclusion.

### Screening and Selection

Two researchers (CB and FR) independently screened the titles and abstracts of all the articles, and the full texts of potentially relevant papers. This gave a moderate inter-rater reliability (κ = 0.42) [24]. Discrepancies were discussed with CM and RW. All qualitative peer-reviewed publications in English exploring barriers faced by adult AS&Rs, or displaced persons to accessing MH services, mental HC delivery, or help-seeking behaviors in HIC were included. Displaced persons were included to ensure all forms of forced displacement were taken into account, including irregular migrants, provided the displacement took place in or to HIC. Books, chapters, dissertations, literature reviews, and theoretical texts were excluded. Articles focusing on individuals under the age of 18 years were also excluded. Studies were also excluded if they did not elicit primary data from participants.

### Assessing Study Quality and Data Extraction

Each paper was individually assessed for quality by author CB using the Critical Appraisal Skills Program (CASP) tool for qualitative studies [25]. A data extraction form was used to summarize bibliographic information, study design, key findings, and limitations. The seven stages of the CF were included in the data extraction process to highlight which study addressed which stage. Author CB read each paper and conducted the data extraction, which was monitored by CM.

### Data Synthesis

A two-stage *critical interpretive synthesis* (CIS) [17, 26] approach was used. In stage one ‘*First order* constructs (i.e. direct quotes used in the papers) and *second order* constructs (i.e. researchers’ interpretations based on existing theories) were identified and merged across studies. This was done by initially extracting all the direct quotes which addressed the themes of accessing and/or negotiating HC from each paper. For example, a first order construct found in a paper published by Teunissen et al. [27] was the quote: ‘*Yeah but we didn’t knew that you can go to a GP with depression*’ (p. 8). The quotes were put in a table together with the second order constructs provided by the original authors of the study. In this case, Teunissen et al. [27] interpreted the quote as demonstrating a lack of recognition and trust of the GP being a doctor who could treat mental illness. The first and second order constructs were compared and contrasted across the different studies through which third order constructs emerged. In this example, the third order construct was ‘understanding a new system’. This process was undertaken by author CB and peer-reviewed by a second researcher (CM).

In stage 2, evidence from across the studies including first, second and third order constructs were integrated into the synthesizing argument, namely the seven stages of the CF. In this example, the first, second and third order constructs mapped onto stage 2 (Navigation). This was peer reviewed by researcher CM and author RW. Overall, a deductive qualitative approach was used.

## Results

**Fig. 1:**
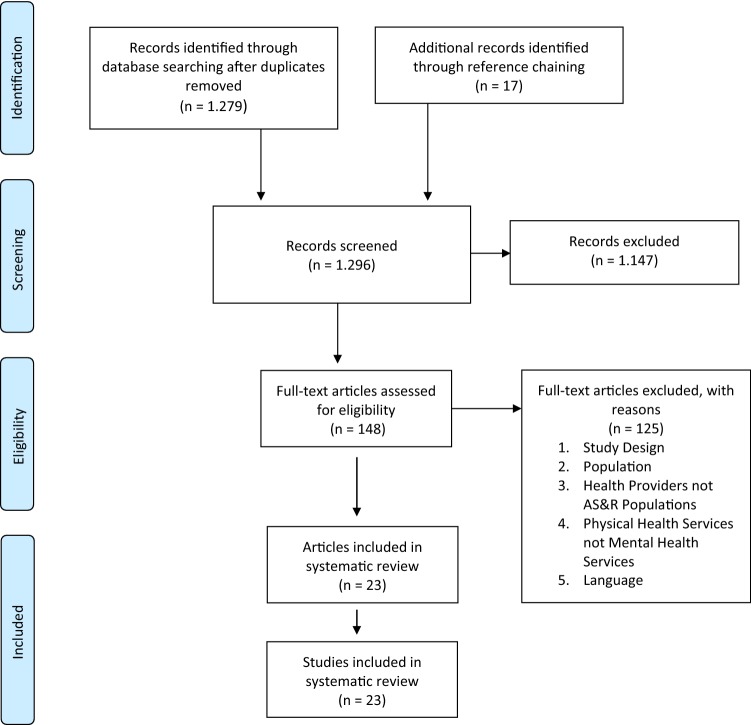
PRISMA flow diagram of the literature search

Of the 1.296 articles identified through the systematic search, 23 met the full inclusion criteria and were included. Article selection is summarized in Fig. 1.

The 23 studies that met the inclusion criteria were conducted in 8 different high-income countries (USA = 3; UK = 4; Canada = 8; Denmark = 1; Switzerland = 1; Australia = 3; Netherlands = 2; New Zealand = 1). Across these studies, 548 participants (Mdn = 21, IQR = 26) were recruited with a representation of 60 different countries of origin. Of 60 participants the specific country was not reported. A summary of the demographic characteristics is shown in Table 2.

**Table 2:** Summary characteristics of studies included in the review

Study	Country study conducted	Participants	Recruitment	Type of migrant	Country of origin	Data collection	Qualitative analysis
Ahmed et al. (2008) [65]	Canada	10 Participants Age 20–40 Gender: female	Purposive sampling	Refugees and asylum seekers	China (2), India (2), Pakistan (1), South America (3), Egypt (1), and Haiti (1)	Semi-structured interviews	Constant comparative method
Ahmed et al. (2017) [35]	Canada	12 Participants Age 20–37	Purposive sampling	Refugees	Syria	Focus groups	Thematic content analysis
Asgary & Segar (2011) [32]	United States of America	35 Participants Age > 40 years Gender: 30 male, 5 female	Purposive sampling	Asylum seekers	Cameroon (4), Chad (4), Guinea (4), Pakistan (3), Bangladesh (2), Congo (2), Kosovo (2), Senegal (2), Sierra Leone (2), Egypt (1), Eritrea (1), Ghana (1), India (1), Ivory Coast (1), Lebanon (1), Mali (1), Mauritania (1), Nepal (1), and Russia (1)	21 semi-structured interviews 5 focus groups	Comprehensive analysis, not specified further
Behnia (2003) [31]	Canada	36 Participants Age 20–49 Gender: not specified	Purposive sampling	Refugees	Bosnia, Cambodia, El Salvador, Iran, and Somalia (numbers not specified)	Semi-structured interviews	Not specified
Campbell et al. (2014) [34]	Canada	21 participants Average age 45.62 Gender: female	Purposive sampling	Refugees, Refugee claimants and undocumented migrants	Mexico (4), El Salvador (2), Colombia (3), Venezuela (4), Ecuador (2), Cuba (1), Dominican Republic (2), Costa Rica (2), South America (1)	Semi-structured interviews	Thematic content analysis
Chase et al. (2017) [20]	Canada	25 participants Average age 36.7, minimum and maximum not provided Gender: 11 males, 13 females, one not specified	Purposive sampling	Asylum seekers	Sub-Saharan Africa (10), North Africa (3), the Middle East (3), South Asia (2), Southeast Asia (1), the Caribbean (5), and South America (1), individual countries not specified	Semi-structured interviews	Thematic content analysis
Djuretic et al. (2007) [66]	United Kingdom	19 participants Age 20–69 years Gender: 7 male, 12 female	Purposive sampling	Refugees, asylum seekers	Croatia (3), Bosnia and Herzegovina (9), Serbia and Montenegro (4), Kosovo (1), Macedonia (1), Slovenia (1)	Focus groups	Thematic content analysis
Donnelly et al. (2011) [67]	Canada	10 participants Age >18 years Gender: all female	Purposive sampling	Refugees	China (5), Sudan (5)	In-depth individual interviews	Framework analysis
Feldmann et al. (2007) [38]	The Netherlands	36 participants Age 18–66 Gender: 15 males, 21 females	Purposive sampling and snowball sampling	Refugees	Afghanistan (36)	Semi-structured interviews	Comparative analysis
Jensen et al. (2014) [39]	Denmark	5 participants^1^ Age 26–50 years Gender: 3 males, 2 females	Purposive sampling	Refugees	Iran (1), Bosnia and Herzegovina (1), Iraq (2), Turkey (1)	Interviews	Thematic content analysis
Kahn et al (2018) [68]^a^	Canada	7 participants Age 22–40	Purposive sampling	Forced migrants (legal status not specified)	Bahamas, Bangladesh, Iran, Lebanon, the Arabian Peninsula, and Ghana (numbers not specified).	In-depth interviews	Thematic content analysis
Leavey et al. (2007) [36]	United Kingdom	9 participants Age 19–41 years Gender: 8 males, 1 female	Purposive sampling	Refugees and asylum seekers	Turkey (8), Cyprus (1)	In-depth interviews	Narrative analysis
Maier & Straub (2011) [69]	Switzerland	13 participants Age 22–53 years Gender: 8 males, 5 females	Purposive sampling	Refugees and asylum seekers	Bosnia and Herzegovina (2), Kosovo (2),Turkey (Turkish) (1),Turkey (Kurdish) (1), Iran (Kurdish, (2), Afghanistan (2), Cameroon (1), Sudan (1), Chechnya (1)	Semi-structured interviews	Thematic content analysis
O’Mahony et al. (2012) [70]	Canada	30 participants Age not specified Gender: females	Not specified	Immigrant (not specified) and refugees	Not specified	In-depth critical ethnographic interviews and field notes	Critical ethnography
Omar et al. (2017) [29]	Australia	36 participants Age 18–60 Gender: males	Purposive sampling	Refugees	Somalia (17), Ethiopia (2), Djibouti (3), Eritrea (6), Saudi Arabia (5), Sudan (2), unknown (1)	Focus groups	Thematic content analysis
Palmer (2007) [28]	United Kingdom	10 participants Age >18 years Gender: 7 males, 3 females	Snowball sampling	Refugees	Ethiopia (10)	In-depth semi-structured interviews	Thematic content analysis
Palmer & Ward (2007) [71]	United Kingdom	21 participants Age 21–62 years Gender: 11 males, 10 females	Maximum variation sampling	Refugees and asylum seekers	Turkey (1), Bosnia and Herzegovina(1), Colombia (1), Democratic Republic of Congo (1), Ethiopia (3), Iran (3), Iraq (2), Kosovo (1), Russia (1), Rwanda (1), Somalia (5), Ukraine (1)	In-depth interviews	Thematic content analysis
Pavlish et al. (2010) [72]	United States of America	57 participants Age 18–80 Gender: females	Purposive sampling	Refugees	Somalia (57)	Focus groups	Inductive coding
Piwowarczyk et al. (2014) [30]	United States of America	48 participants Age 18–59 years Gender: all female	Convenience sample	Refugees and asylum seekers	Democratic Republic of Congo, Somalia (numbers not specified)	Focus groups	Grounded theory
Shrestha-Ranjit et al. (2017) [73]	New Zealand	40 participants^a^ Age 18–82 Gender: 8 males, 32 females	Not specified	Refugees	Bhutan (40)	Focus groups	Thematic content analysis
Russo et al. (2015) [33]	Australia	38 participants Age > 18 years Gender: all female	Purposive sampling	Refugees	Afghanistan (38)	In-depth interviews and focus groups	Thematic content analysis
Teunissen et al. (2014) [27]	The Netherlands	15 participants Age 21–73 years Gender: 9 males, 6 females	Purposive sampling	Undocumented migrants	Burundi (1), Dominican Republic (1), Egypt (1), Eritrea (1), Ghana (1), Morocco (1), Nepal (1), Nigeria (1), Philippines (2), Sierra Leone (1), Somalia (1), Surinam (1), Uganda (1), Zambia (1)	Interviews	Grounded theory
Valibhoy et al. (2017) [37]	Australia	16 participants Age 18–25 years Gender not specified	Purposive sampling	Refugees	Iraq, Iran, Afghanistan, Sudan, Democratic Republic of Congo, Ethiopia, Tanzania, Ivory Coast, Pakistan (Numbers not specified)	In-depth individual interviews	Thematic content analysis

^a^Only the answers of AS&R participants were included in this review

**Fig. 2:**
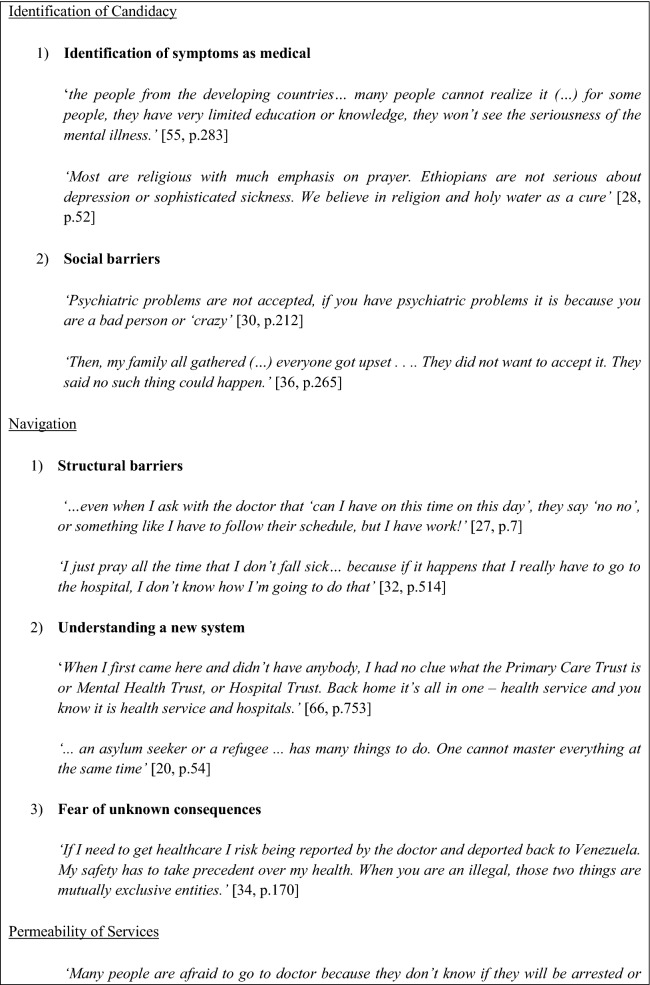

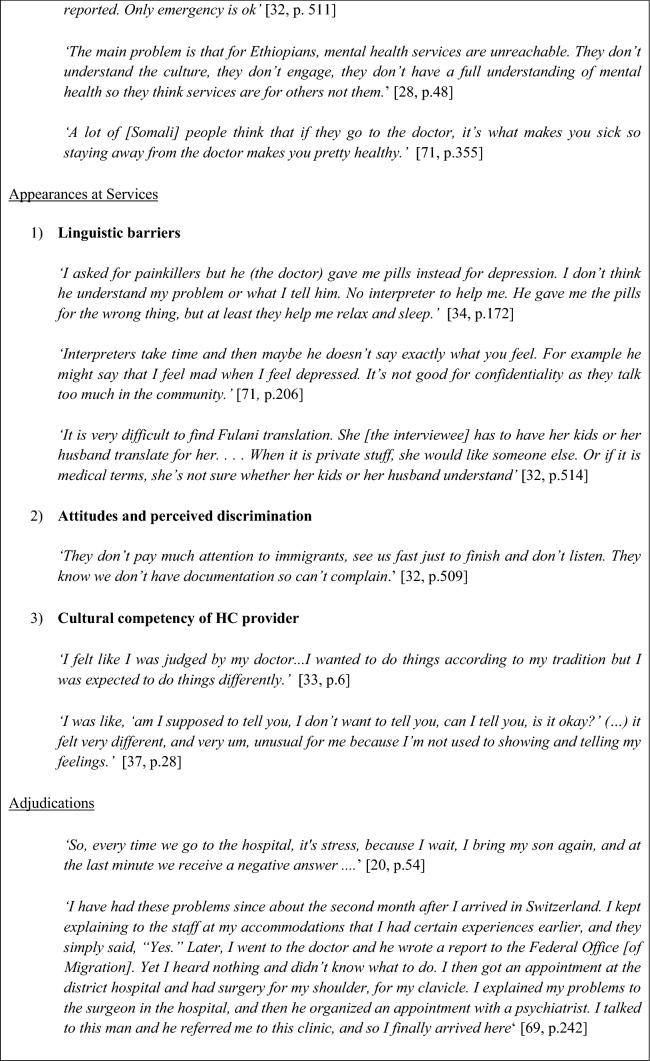

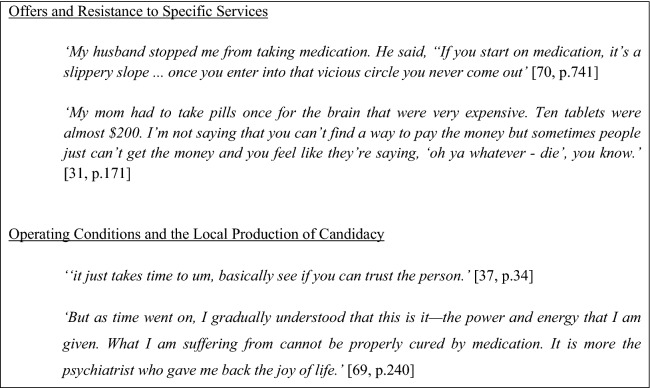
Key thematic quotations

Table 3 provides an overview of which stage(s) of candidacy were addressed by each study. All studies addressed at least 2 stages, the *Identification of candidacy* (stage 1) was the most widely discussed by 20 studies and *Adjudications by Professionals* (stage 5) was the least commonly discussed, reported on by only 7 studies. Additional quotes to support the findings for each stage are included in Fig. 2.

**Table 3:** The stages of candidacy addressed by studies (N=23)

Article	Stage 1	Stage 2	Stage 3	Stage 4	Stage 5	Stage 6	Stage 7
Ahmed et al. (2008) [65]	✓	✓		✓			✓
Ahmed et al. (2017) [35]	✓	✓		✓			
Asgary and Segar (2011) [32]	✓	✓	✓	✓			✓
Behnia (2003) [31]	✓					✓	✓
Campbell et al. (2014) [34]		✓	✓	✓		✓	✓
Chase et al. (2017) [20]		✓	✓	✓	✓		
Djuretic et al. (2007) [66]	✓	✓		✓	✓		
Donnelly et al. (2011) [67]	✓	✓	✓	✓		✓	✓
Feldmann et al. (2007) [38]				✓	✓	✓	✓
Jensen et al. (2014) [39]	✓	✓			✓	✓	✓
Kahn et al. (2018) [68]	✓	✓					✓
Leavey et al. (2007) [36]	✓			✓		✓	
Maier and Straub (2011) [69]	✓				✓	✓	✓
O’Mahony et al. (2012) [70]	✓	✓				✓	✓
Omar et al. (2017) [29]	✓	✓	✓				
Palmer (2007) [28]	✓		✓				
Palmer and Ward (2007) [71]	✓		✓	✓			✓
Pavlish et al. (2010) [72]	✓		✓			✓	
Piwowarczyk et al. (2014) [30]	✓					✓	
Shrestha-Ranjit et al. (2017) [73]	✓		✓	✓	✓		
Russo et al. (2015) [33]	✓	✓		✓			
Teunissen et al. (2015) [27]	✓	✓	✓	✓		✓	
Valibhoy et al. (2017) [37]	✓	✓		✓	✓	✓	✓

Stage 1= Identification of candidacy, Stage 2= Navigation, Stage 3= Permeability of services, Stage 4= Appearing at services and asserting candidacy, Stage 5= Adjudication by professionals, Stage 6= Offers of and resistance to specific services and Stage 7= Operating conditions and local production of candidacy

### Identification of Candidacy

The identification of candidacy was dependent on two different third order constructs.

#### Identification of Symptoms as Medical

Different studies found that AS&Rs often did not seek medical help because they were not familiar with symptoms of mental illness, they did not consider the seriousness of their symptoms, or there was a cultural difference regarding the interpretation of symptoms. Traditional beliefs around symptoms being caused by supernatural forces such as cursing, witchcraft or evil spirits led many individuals to describe people with mental illness as deserving of their condition. Many studies also showed that individuals commonly believed the illness to be part of their destiny, therefore participants often relied on alternative forms of care and even described a lack of awareness of formal services to provide support.

‘Traditionally it is believed that diseases can be caused because of cursing, and by evil spirit and germs. The remedies are medicinal plants, praying, healers and taking tablets.’ [28, p. 51]

Alternative forms of care were largely traditional practices, which were mentioned as a source of support and strength to deal with MH symptoms. These included healing through the church, herbal remedies, praying or going to ceremonies. Prayer was most commonly reported across studies as a valid coping method and a good alternative to formal care. Individuals mentioned prayer as bringing relief and calm, thus helping to cope with MH symptoms. Findings highlighted that traditional practices were mostly supported by older generations, with younger individuals at times preferring formal services in the country of resettlement. Family pressures influenced these decisions, as parents often pushed younger generations to use these practices despite the youngsters not believing in their effectiveness.

‘For our old generation, if someone is sick we quickly invite Sheikh to read Quran on him and I don’t think that young people use Quran as a healing (…) as far as I know, some, their parents beg them to accept reading Quran on them…’ [29, p. 384]

Other forms of care included relying on personal and easily accessible resources rather than seeking external help in dealing with stressful situations. Social networks from the country of origin were seen as a valid form of counselling, specifically accessing shared environments that held a common language, culture, and history. One individual explained; ‘*Counsel in each other. Look to friends, family, religious leaders*’ [30, p. 212]. These ideas mostly emerged amongst individuals who were not familiar with or distrusted the local HC system, with distrust often stemming from alternative ideas around the causation of illness and other barriers discussed in stage 2 (navigation). These findings highlight that although individuals may identify their candidacy; they are often choosing not to access formal services.

#### Social Barriers

Social barriers to identifying candidacy included stigma and privacy concerns surrounding MH. Stigma emerged both from the individuals themselves and from the environment and was often embedded in preconceived ideas of what individuals with mental illness are like. At times, the individuals appeared to internalize the stigma as shame, hindering the willingness to ask for help for fear of social stigma. The environment also discouraged individuals from seeking care particularly family pressures and gender hierarchy influenced whether entitlement and need for help was recognized or not. For example, at times male domination situated women in a socially vulnerable position, thereby hindering timely identification of candidacy. There were also worries around confidentiality, given that if services were accessed, confidentiality could not be ensured which may lead to further stigmatization from the community.

‘I don’t use mental health professional. In my culture going to a professional like a psychologist and psychiatrist is stigmatized. It is associated with mental health problem and craziness.’ [31, p. 12]

### Navigation

Using services was dependent on three different third order constructs.

#### Structural Barriers

Location of the medical center, inability to pay for transport, cancelling work for the appointment, and finding someone to look after the children, were all found to be structural barriers to navigating HC systems. Preoccupations with medical expenses interfered substantially with seeking care and choice of service, especially when host countries required medical insurance. Participants also felt that providers often did not understand their situation in terms of economical options of paying for treatment and some voiced their mistrust of Western biomedicine altogether stating that providers are only after money.

“I just got this temporary job and my boss would not allow me to leave to see doctor” [32, p. 515]

#### Understanding a New System

The ability to navigate a new and unfamiliar system was mostly dominated by a lack of knowledge about the right to medical HC and where and how to attain it. There were notions that initially participants believed they were entitled to certain forms of HC but were unclear on the scope and duration of the coverage. AS&Rs who had arrived individually or did not speak the local language described it as a time-consuming process to understand routes to accessing primary care. Specifically, understanding the ‘gatekeeper’ role of primary care services. Furthermore, many AS&Rs reported not being familiar with the actual role of MH professionals, nor what symptoms could be treated. Participants addressed the need for information to be made available when arriving to the host country, specifically which services exist and what they charge.

‘Yeah but we didn’t knew that you can go to a GP with depression, we didn’t know that.’ [27, p. 8]

#### Fear of Unknown Consequences

Fear of unknown consequences to accessing services was common. There was a pervasive fear of approaching authority of any kind and facing legal consequences. Personal safety was often chosen over health, especially with undocumented migrants. Preoccupation existed around health-care-related bills and fear regarding inability to pay and consequently being reported to authorities. Additionally, there was a fear that receiving a MH diagnosis would result in separation from family or children.

“I also had the fear that if I talked to someone that people will come and take my daughter from me because I thought I was going crazy.” [33, p. 6]

### Permeability of Services

Often influenced by the previously mentioned barriers, some individuals delayed seeking medical help until reaching a crisis point and thereby accessing urgent care. This was particularly the case for undocumented migrants. This delay was at times also influenced by previous experiences, where individuals described a substantial gap between initial refusals and further help-seeking behavior. Initial refusals influenced the permeability of services in that participants felt they were not taken seriously when reaching out for help. If past experiences were positive, individuals were more willing to establish their candidacy again.

‘If I get sick I pop pills and wait. And when I say pills I mean over the counter shit, not prescriptions. If it gets really bad then I have to decide if I think I will die. If I think I will, I go to Emergency. If I don’t then I wait in pain. Why do you ask me about family doctors? Walk-in-clinics? Are you kidding? I have no papers.’ [34, p. 171]

Economic worries also restricted permeability, and many individuals described learning about their entitlements on a trial and error basis: *“… I do not know [if it will be covered] until I try, when I go*” [20, p. 54]. The willingness to do so was described as dependent on whether it was themselves or their children who were in need of care, with there being less hesitation when it concerned a child.

Continuity of care, sub-specialties, and preventive care were largely unknown or unavailable to users. Importantly, they often depended on a range of support services such as non-governmental organizations to access care and be referred to specialist care. Additionally, there also appeared to be cultural norms which influenced whether services were seen as permeable. Evidence showed issues with religion, language, and expectations on what would happen if they did, for example many participants expressed that they believed that going to the doctor is what makes you sick therefore staying away keeps you healthy. These barriers highlight the lack of knowledge and therefore the lack of permeability of specialist services for AS&Rs.

### Appearances at Health Services

Barriers when appearing at services were linguistic; attitudes and perceived discrimination; and cultural competency.

#### Linguistic Barriers

Language was a major obstacle when appearing at health services, with a lack of adequate translation services, particularly for uncommon languages, found across studies. This was often linked to fear, as inability to communicate with providers led to uncertainty around outcomes. Confidentiality was a big issue, as worries were voiced regarding official interpreters and their role in the community. Furthermore, studies found concerns around interpreters them omitting material, interpreting inaccurately, hindering interpersonal dynamics, giving opinions, or even passing judgment. Alternatively, family members were sometimes used for translational purposes, but this brought up issues of confidentiality of its own.

“If you speak with a psychiatrist, you would speak normally, but if there is an interpreter as a mediator, and this person might speak about what you said, and now like you have told your story to this and may be this mediator will tell everybody in Canada.” [35, p. 8]

#### Attitudes and Perceived Discrimination

Studies reported that the attitudes and perceived discrimination at HC services led to feelings of rejection, especially when participants felt their concerns were not taken seriously, were disregarded by HC professionals, or they felt they were treated differently from the national citizens. This was mostly prominent when participants felt they received hostile attitudes when they used their immigration papers or lacked insurance.

‘For me, it’s moral torture…. Sometimes I pray God to give us good health, me and my children, because I know so well what I will face in clinics or in hospitals…. You feel worth less than others, as if you don’t have the same rights as the other person.’ [20, p. 55]

A sense of discriminatory attitudes also occurred when there was a cultural misalignment in terms of the symptomology description. Some individuals found it difficult to talk about their experiences or feelings with someone unfamiliar to them, therefore resorting to the use of subtle terms to describe symptoms. Individuals felt that this sense of unease lead some HC providers to conclude that the situation was not serious, or they focused on a different illness altogether. One study highlighted a service user who described feelings of embarrassment when during his first visit the practitioner had begun to ask ‘inappropriate’ questions relating the HIV/AIDS and tuberculosis, which were not the reason for the visit [27]. This led to feelings of discrimination, as the individual felt these questions were only due to his immigration status.

#### Cultural Competency of HC Provider

Several studies discussed how care conflicted with cultural practices of the individuals, and this was identified as a point of contention in their appearance at services. For example, many AS&Rs were unaccustomed to verbalizing personal experiences and emotions. One study highlighted an individual who had experienced ‘spiritual energies’ since he was seventeen and viewed his problems mostly in religious terms [28]. He indicated that he would not discuss these issues with English doctors as they would not understand these terms and spiritual beliefs—‘*they are only interested in symptoms*.’ [36, p. 264]. This was highlighted as feelings of judgment from the providers for wanting to adhere to their own traditions. It was considered important that health workers recognized these beliefs as being legitimate and culturally significant.

### Adjudications by Professionals

Findings showed that once AS&Rs had asserted their candidacy by presenting to health services, the professional judgements made regarding their candidacy strongly influenced subsequent access to services. AS&Rs highlighted a lack of resources and inconsistencies between providers. Providers were often perceived as overwhelmed with few options for referring clients who required continuing MH care. Programs designed for AS&Rs often lacked funding or were oversaturated with long waiting times. This resulted in concerns about timely access to a specialist’s opinion. It was however generally recognized that severe conditions were referred more quickly. Respondents also mentioned complex referral processes and eligibility criteria for accessing MH services leading to negative experiences and absence of clear guidance as to how to navigate the system.

“doctors at [A] they suggested [B]. I contacted [B] and then they couldn’t do help much. Then I was transferred to [C] and from there to [D] so it’s like a little tour.” [37, p. 29]

Service users also described experiences of being turned away from the service if they were not assessed to be ill enough when they reached out for help. The lack of referrals led to feelings of rejection by the system and AS&R experienced that they often had to rely on other people to negotiate contact with the services on their behalf in order to be taken seriously.

Cultural concerns with regards to adjudication also emerged. For example, concerns regarding whether HC providers in the country of resettlement were familiar with the common diseases in their countries of origin. Another example that arose was the feeling amongst participants that an illness should be treated in the early stages rather than waiting to see the symptoms develop *‘they let your illness become very old here’* [38, p. 522].This was further underscored by the idea that health services may lack staff that is knowledgeable and sensitive to the particular needs of AS&R populations.

### Offers and Resistance

This stage was dominated by concerns over an excessive focus on medication. Experiences of emphasizing watchful waiting approaches and simple self-medication, was perceived by some as revealing a lack of interest in them and their circumstances. Simultaneously, when prescribed medication, there was a lack of understanding what it was for and worries around consequences such as addiction, side effects, or medication leading to worsening of the problem emerged across studies. Mostly, these findings suggested a lack of communication between the provider and patient regarding the purpose of the medication.

‘Sometimes I see these pills… I mean I don’t think that these pills are good. They make me numb. Sometimes I decide to give up. I decide to skip taking them to see what would happen.’ [36, p. 263]

A few individuals in the studies described their statements and behaviors as being exaggerated to fit within a particular illness framework, making it more difficult for them to come to terms with their diagnosis and accept treatment offers. The lack of acknowledgement of the individuals’ perspective towards treatment and their past experiences led to patients feeling detached from their treatment course. In some cases, this resulted in individuals reducing or discontinuing their medication without the involvement of the health professional. This was at times also influenced by the environment including family and clergy, who even if the medications were prescribed sometimes discouraged the individual from taking them. Often this was prioritized over treatment, and the disparity between lay beliefs and Western understanding of MH created a clash of understanding between the service user and the system.

‘If a doctor says you need medication, and the pastor says no. You won’t take it’ [30, p. 212]

Issues around the economic burden of medication also emerged as a reason for resistance and discontinued treatment. Out-of-pocket payment proved to be challenging especially for those who do not have the finances to cover food and housing expenses.

### Operating Conditions and the Local Production of Candidacy

The relationship with the individual provider was highlighted as being essential to the continued use of services. Specifically, trust, rapport and respect in the relationship were key, and suggested that satisfaction with operating conditions and production of candidacy were very person dependent and could take years before they found a provider with whom they developed a deeper connection.

‘Sometimes they were asking very like personal questions that I didn’t like…The journey that we had, like how many days were you in the boat, and I never want to think about it… [later] People are different, like we have saying; ‘‘jungle has dry and wet—some trees are alive, some trees are dead, and they are different.’’ And people are the same; some people like to talk about their selves, their families, and some people want to keep a secret.‘[37, p. 32]

There were also instances in which individuals had not been able to build this relationship which often led to discontinuing the care. The main reason for this was providers not meeting expectations or not being adequately responsive to needs. A need for awareness of the individuals’ cultural background, previous experiences, and understanding how the individual made sense of their illness was very salient. Findings showed the need for practitioners to avoid assumptions and learn from the patient as an individual in order to accommodate nuances in ethnic and religious identities. For example, one female refugee who had previously been incarcerated in Iraq highlighted her distress upon being in a closed ward showing the need for providers to understand the individual’s past: *‘And the door was shut. It was a closed ward. It comes to my mind again, how we were in prison in my country. The door was shut. It was very difficult.*’ [39, p. 9749].

Lastly, studies reported accounts of professionals being out of reach due to a lack of time giving rise to feelings of neglect, unworthiness, and frustration in patients. Furthermore, the flexibility of services to respond to individual needs was highly valued. This included adapting the frequency of consultations to personal preferences or maintaining contact with the provider whilst waiting to access specialized treatment.

“he was not helpful or he was just not getting us… I felt he was being disrespectful… we were new to the country and… we had to travel by train then take a tram and sometimes we might be a bit late, but he wasn’t understanding one bit.” [37, p. 35]

## Discussion

The current review used the candidacy framework (CF) to synthesize qualitative findings relating to barriers to accessing and negotiating MH services for AS&R in high-income countries. Comparatively more data was available in the papers about barriers to access than on barriers to negotiating services once accessed. This could be an artefact of the fact that barriers to accessing services mean that a small number of respondents can comment on issues relating to negotiating services, or that this has been less of a focus of research conducted to date. Nonetheless, findings show there are many barriers which affect the process of establishing candidacy for care which affirm the harmful consequences of barriers including delays in receiving treatment, feelings of social exclusion and mistrust.

### Access to Services

The identification of candidacy (stage 1) was dominated by issues relating to the interpretations by AS&Rs of symptoms and social barriers. The data showed that AS&R recognize their symptoms as requiring help, however they often turn to informal services. Previous evidence has suggested that traditional explanatory models of health held by ethnic minority groups can impact on their help seeking behavior from Western HC services. This may be attributable to different explanatory models regarding MH, specifically different holistic beliefs about causality that do not correlate with the western medical model [40]. Certain beliefs about causality can lead directly to shame and stigma, such as MH problems as punishment for wrong-doings [41], as God’s will, and as black-magic, jinn or possession by spirits [42, 43]. On the other hand, in a qualitative study conducted on a Thai Muslim community, family and key stakeholder participants rejected the idea that schizophrenia had stigma since the illness was Allah’s will [44]. Consequently, individuals may choose to access more traditional and faith-based healing practices [45], as was found in the current review. Access to services may not be sufficient, it must be accompanied by efforts to increase MH literacy for communities and training for traditional and faith-based healers to improve referral pathways to formal services and decrease stigma.

The concerns over structural barriers (i.e. fear of financial contribution) and unknown consequences (i.e. legal repercussions) to accessing services found in stage 2 (*navigation)* suggest that there is a clear need to provide more knowledge on available services and entitlements to care in this population across Western countries. The unknown consequences of accessing services combined with an inherent lack of trust in public organizations and/or fear of being reported to authorities can make it particularly challenging for individuals to trust HC systems especially during the asylum process [46]. Their migratory status has the potential to perpetuate social dependence and economic marginalization [47] and therefore hindering their assertion to candidacy and accessing health services.

### Negotiating Services

The negotiation stages highlighted the dynamic nature of the system and more specifically the constant negotiation between service users and HC providers. Overall, service-level responsiveness was inadequate with waiting lists, eligibility criteria, and continuity of care being described as common and distressing. The findings suggest that power distributions were asymmetrical at times between HC providers and AS&R including the enforcement of dominant values onto services users and perceived discrimination.

Theorists commenting on the difference between illness and diseases have emphasized ‘illness’ as the individual’s lived experience of symptoms and disability; and ‘disease’ as the HC provider’s representation of the disorder after having reworked the person’s account into a medical framework [48, 49]. Understanding how individuals create meaning in their illness can largely influence care and increase diagnostic validity [50, 51]. This calls for the need for culturally competent care, which exists when providers are knowledgeable of the potential and actual factors that can influence their interaction with service users and have training to address the cultural divide [52]. However, dominance of the bio-medical model may fail to adequately acknowledge the social and cultural basis of MH. Providers can be influenced by stereotypes and potentially homogenize this population into a single pathologized identity, or lack training to identify symptoms unique to other cultures [53]. Therefore, providers must constantly reflect on their own values, attitudes and behaviors that could be influencing the relationship and can both directly and indirectly create barriers to care [54–56].

Language was flagged as a major barrier throughout the current review. In terms of access, individuals were scared providers would not speak their language or understand their symptoms. In terms of negotiating the system, language existed as a barrier throughout the stages. The lack of competent interpretation was said to complicate the encounter and translational services were often not available for comparatively rarely spoken languages and dialects. When this occurred, providers often used family members as translators, which highlight suboptimal standards as this has implications for potential bias in the interpretation, and reduced willingness on the AS&Rs’ behalf to open up. MH providers themselves have also reported similar issues including lack of access to or poor-quality interpretation services in research [57, 58]. This has been found to impact empathetic responses, decrease rapport, service user satisfaction and has shown to increase medical error in previous research [59, 60]. The sensitive nature of AS&Rs’ experiences demands highly competent interpretation services therefore there is a need to train clinicians systematically in the efficient use of interpreters, cultural brokers and cultural formulations as has been highlighted previously [61, 62]. Additionally, interpreters may require additional training to work with AS&R and clinicians in what may be challenging consultations. Piacentini et al. [63] have previously highlighted the need for more training measures that move beyond diversity and/or race awareness, and which use a more holistic approach to understanding how different social identities and multi-dimensional markers of difference come to be produced and reproduced in interpreter-mediated healthcare encounters with migrant populations. They argue that these social identities and markers of difference include language, culture, ethnicity, age, gender, and also immigration status. Therefore, interpreters need to be aware how these variables intersect specifically with language.

The use of the CF as synthesizing argument for CIS has proven to be a useful way to conceptualize barriers and underlying constructs that influence access and negotiation. Using a systematic review to bring this knowledge together has allowed us to cast the net wide and integrate findings from different global settings into new evidence-based knowledge.

### Recommendations for Improving Practice

Moving forward, a holistic approach incorporating input from a range of stakeholders is needed to address the barriers found in this review, including the work of academics; policy makers and HC providers who all need to acknowledge the impact of country of origin, language, culture and status on MH service provision. Most importantly, the idea that ‘one size does not fit all’ should be at the forefront. Once service users have accessed mainstream health services, simple referral processes and provision of adequate information can facilitate treatment, for example through websites [46, 62]. Furthermore, sensitivity trainings, hiring professionals who share the persons’ ethnicity or language, and improvement of interpretation services are needed. Additionally, interpreters may require additional training to work with AS&R and clinicians in what may be challenging consultations. Piacentini et al. [63] have previously highlighted the need for more training measures that move beyond diversity and/or race awareness, and which use a more holistic approach to understanding how different social identities and multi-dimensional markers of difference come to be produced and reproduced in interpreter-mediated healthcare encounters with migrant populations. They argue that these social identities and markers of difference include language, culture, ethnicity, age, gender, and also immigration status. Therefore, interpreters need to be aware how these variables intersect specifically with language. Most importantly, AS&Rs need to be engaged as stakeholders and stand at the center of finding solutions to achieving accessible and negotiable services.

There is also a need for qualitative research into displaced populations’ barriers to HC in low and middle-income countries. This review focused on high-income countries but can be seen as examples of the types of issues that local MH services should be exploring with their own AS&R communities. The CF has thus far only been used in high-income settings, therefore future research should investigate the suitability of using the CF in low and middle-income settings where more macro level barriers to care may exist. Lastly, this review only considered barriers to access and negotiation rather than including facilitators. There is a need for future reviews to address facilitators that can increase contact with services.

## Strengths and Limitations

This review was the first to focus specifically on barriers to MH services for AS&Rs by using a CF. The use of qualitative research afforded opportunities for the personal experiences of AS&Rs to be explored in depth. Given the cultural diversity of the sample, these findings appear to be generalizable for AS&Rs who migrate to Western countries despite varying national policies and HC systems in their countries of resettlement.

Regarding the CIS, accessing first order constructs (i.e. participants in the research) was not possible as the data included in the primary studies had already been preselected from initial datasets. For this review, second order constructs (i.e. researchers’ interpretations of these views based on theories) were arguably more representative of the overall findings relating to barriers. This made it difficult to distinguish the influence of authors’ perspectives in terms of personal background or theoretical standpoints. Additionally, the use of translators in the studies entails a potential omission of information and/or errors in the translation process, which makes this distinction complicated. The strength of CIS is that it can link the emerging synthetic constructs surrounding barriers to access and negotiation to the chosen synthesizing argument of candidacy. This theoretical framework further allowed the transition from simply describing the barriers to understanding the multidimensional nature thereof.

In terms of generalizability, all studies included were based in high-income countries. Given that the majority of the world’s AS&R live in low and middle-income countries [64], this limitation highlights the importance of further research concerning barriers to accessing and negotiating care for AS&Rs in low and middle-income settings.

## Conclusion

The findings of this review reflect a rich experience of barriers to accessing and negotiating MH services for AS&Rs. By doing so it has begun to unpack and differentiate the unique barriers to MH care faced by these groups, as opposed to a more broadly defined ‘immigrant’ or ‘foreign-born’ population. The use of the CF provided a theoretical framework to understand the inter-related barriers, which exist at different stages. Reduced access ultimately leads to decreased health status and increased suffering amongst a population at elevated risk of experiencing MH difficulties. The CF has proven to be effective for gaining insight into barriers and the necessary refocusing of future research, policy and practice to ameliorate these barriers. The bio-medical model may not be a sufficient service model for meeting AS&R MH needs, with more focus needed on non-health sector interventions with more inclusive explanatory models.

## Appendix A—Search Strategy for PsycINFO

1. “Political Asylum” OR “Refugees” OR “Asylum Seeking” OR “Displaced Person”
2. (Asylum N2 Seek*) OR (refuge*) OR (displaced N1 person*) OR (Political N1 Asylum)
3. S1 OR S2
4. “Community Counseling” OR “Community Mental Health” OR “Community Psychology” OR “Mental Health” OR “Mental Health Services” OR “Community Mental Health Services” OR “Community Psychiatry”
5. (Communit* N1 counsel*) OR (Communit* mental N1 health) OR (Communit* N4 psych*) OR (Mental N1 health) OR (Mental N1 health N1 service*) OR (mental health OR Psycholog*) N4 (service* OR cent* OR care)
6. “Adjustment Disorders” OR “Affective Disorders” OR “Anxiety Disorders” OR “Dementia” OR “Dissociative Disorders” OR “Eating Disorders” OR “Impulse Control Disorders” OR “Mental Disorders due to General Medical Conditions” OR “Neurosis” OR “Personality Disorders” OR “Pseudodementia” OR “Psychosis” OR “Behavior Disorders” OR “Borderline States” OR “Brain Disorders” OR “Chronic Illness” OR “Comorbidity” OR “Conduct Disorder” OR “Emotional Adjustment” OR “Emotional Disturbances” OR “Memory Disorders” OR “Organic Brain Syndromes” OR “Perceptual Disturbances” OR “Personality Processes” OR “Sleep Disorders” OR “Suicide” OR “Thought Disturbances” OR “Mental Disorders”
7. “Adjustment N1 Disorder*” OR “Affective N1 Disorder*” OR “Anxi* N1 Disorder*” OR “Dementia” OR “Dissociative N1 Disorder*” OR “Eating N1 Disorder*” OR “Impulse N1 Control N1 Disorder*” OR “Mental N1 Disorder* due to General N1 Medical N1 Condition*” OR “Neurosis” OR “Personality N1 Disorder*” OR “Pseudodementia” OR “Psychosis” OR “Behavi* N1 Disorder*” OR “Borderline N1 Stat*” OR “Brain N1 Disorder*” OR “Chronic* N1 Ill*” OR “Comorbid*” OR “Conduct N1 Disorder*” OR “Emotional N1 Adjustment” OR “Emotion* N1 Disturb*” OR “Memory N1 Disorder*” OR “Organic N1 Brain N1 Syndrome*” OR “Perceptual N1 Disturbance*” OR “Personality N1 Process*” OR “Sleep N1 Disorder*” OR “Suicid*” OR “Thought N1 Disturbance*” OR “Mental N1 Disorder*”
8. (4 OR 5)
9. (6 OR 7)
10. (8 AND 9)
11. “Help Seeking Behavior” OR “Self-Referral” OR “Health Care Seeking Behavior” OR “Health Care Utilization” OR “Treatment Barriers” OR “Health Care Delivery” OR “Candidacy”
12. (Acces* OR Util*) OR (navigati* of service*) OR (Candida*) OR (Help N1 Seeking N1 Behavi*) OR (Health N1 Care N4 Seek* N2 Behavi*)
13. (11 OR 12)
14. (3 AND 10 AND 13)
